# Association between nutritional status and children and adolescents’ dental caries experiences: an overview of systematic reviews

**DOI:** 10.1590/1678-7757-2023-0138

**Published:** 2023-09-25

**Authors:** Thaís de Oliveira FERNANDES, Patrícia Arriaga CARVALHO, Fernanda Volpe de ABREU, Christian KIRSCHNECK, Erika Calvano KÜCHLER, Leonardo Santos ANTUNES, Lívia Azeredo Alves ANTUNES

**Affiliations:** 1 Universidade Federal Fluminense Instituto de Saúde de Nova Friburgo Programa de pós-graduação em odontologia Nova Friburgo RJ Brasil Universidade Federal Fluminense, Instituto de Saúde de Nova Friburgo, Programa de pós-graduação em odontologia, Nova Friburgo, RJ, Brasil.; 2 Universidade Federal Fluminense Faculdade de Odontologia Programa de pós-graduação em Odontologia Niterói RJ Brasil Universidade Federal Fluminense, Faculdade de Odontologia, Programa de pós-graduação em Odontologia, Niterói, RJ, Brasil.; 3 University of Regensberg Department of Orthodontics Regensberg Germany University of Regensberg, Department of Orthodontics, Regensberg, Germany.

**Keywords:** Dental caries, Nutritional status, Child, Adolescent, Systematic review

## Abstract

An increasing number of systematic reviews (SR) has investigated the association between dental caries and nutritional status in children and adolescents, thus requiring an overview to compile the information in a single piece of evidence. Therefore, this study aimed to evaluate and summarize evidence from published SR on the association between dental caries and nutritional status in children and adolescents. A wide search was conducted on May 29, 2023, in six databases (Medline via PubMed, Scopus, Web of Science, Cochrane library, Embase, and the Virtual Health Library – VHL). An additional search was performed in the gray literature (Open grey and Google Scholar), SR registration databases, and the list of references of the included SR. Our inclusion criteria were based on acronym PECOS. Overall, two reviewers independently extracted the data, evaluated the risk of bias (ROBIS), and assessed the quality of the chosen studies (AMSTAR-2). Data from the included meta-analysis were summarized and certainty of evidence using the GRADE approach was performed. After removing duplicates and applying our eligibility criteria, 19 SR from 2006-2022 were included. We found that 17 SR showed high risk of bias and critically low methodological quality. We observed an association between dental caries experiences and nutritional status since seven SR found an association between obesity/overweight and dental caries; one, an association between underweight and dental caries; and eleven, no associations. The meta-analysis showed divergent results according to the study designs, used indices, and participants’ age group, and were scored as having a very low certainty of evidence. Therefore, based on the high risk of bias, low methodological quality, and very low certainty of evidence of the chosen SR, most studies found no association between children and adolescents’ nutritional status and dental caries experience.

## Introduction

Dental caries is a chronic multifactorial condition that involves individual factors and socioeconomic characteristics and is considered a worldwide public health problem.^1^ The prevalence of this condition in primary and permanent dentition in children and adolescents is high.^2^ Financially, dental caries represents a great investment in health interventions, thus requiring strategies that include educational programs to promote health and prevent this condition, especially for healthcare providers, parents, educators, children, and teenagers.^2^

Nutritional status alterations are public health problems stemming from an imbalance in the consumption of macronutrients or micronutrients, constituting either overnutrition (overweight/obesity) or undernutrition (underweight). This worldwide pandemic has been found as the main cause of the reduction of health standards in children and teenagers,^3^ affecting populations around the world in different manners. Historically, the most widespread condition of nutritional status alteration was underweight, so UNICEF estimated that over 200 million children under five years of age were undernourished.^3^ In contrast, the global prevalence of obese or overweight children and teenagers aged 5–19 years increased from 4% to 18% from 1975 to 2016.^4^

Different nutritional status during childhood and adolescence are associated with long- and short-term negative consequences.^5^ Chronic malnutrition can enhance the cariogenic potential of fermentable carbohydrates in children.^6^ Evidence suggested that undernourished individuals have different levels of dental caries than those with normal/ideal weight.^7^ Several researchers have suggested and investigated the association between obesity and/or overweight with dental caries experiences in different populations since these conditions share similar etiological factors, such as diets high in sugar.^8-10^ Socioeconomic and behavioral characteristics are common factors for dental caries and obesity or overweight as a diet rich in fermentable carbohydrates offer risks for obese/overweight children and teenagers and configures a key factor for dental caries. Managing nutrition variety and quality is crucial to the development of both conditions.^2,4^

Systematic reviews (SR) have been conducted to evaluate the association between nutritional status (underweight, normal weight, overweight, and obesity) and dental caries experiences in children and teenagers, but these studies still lack a consensus,^8-10^ thus requiring the grouping of results by overviewing systematic reviews to reach single evidence as it could improve the aim of public health policies and strategies. Therefore, this study aimed to evaluate evidence from published systematic reviews to summarize the evidence on the association between children and adolescents’ dental caries experiences and nutritional status.

## Methodology

This overview was registered on the PROSPERO database (CRD42022308325). The Cochrane collaboration guide^11^ was followed and this study was written according to the guidelines of the Preferred Reporting Items for Overviews of Reviews (PRIOR).^12^ This overview was performed based on the following question: “Is children and adolescents’ nutritional status associated with dental caries experiences?” A PECO framework (i.e., Population, Exposure, Comparison, Outcome, Study design) was used to structure our search strategy and devise our guiding question and related search terms.

### Search Strategy and article selection process

The literature was searched on May 29, 2023. Medline via PubMed, Web of Science, Cochrane Library, Virtual Health Library (VHL) — to access LILACS (the Latin American and Caribbean Health Sciences Literature) —, Embase, and Scopus were assessed. MeSH terms (Medical Subject Headings) for PubMed, DECS descriptors for VHL, and Emtree terms (Embase Subject Headings) for Embase were combined in our search strategy. Free terms with Boolean operators (OR, AND) and synonyms related to the main outcomes (dental caries experiences and nutritional status), the population of interest (children and adolescents), and the type of study (systematic reviews) were also used. SR protocol registration such as PROSPERO, Open Science Framework, and SR (Epistemonikos) databases was accessed. An additional search was made on the gray literature using Open Grey (http://www.opengrey.eu) and Google Scholar (https://scholar.google.com.br/). The references of the included studies were manually checked for additional studies. The search strategy is shown in Supplemental Table 1.

The SR found in these searches were directly exported from the electronic databases to the Mendeley^®^ Desktop reference manager to remove duplicates and apply our eligibility criteria.

### Eligibility criteria and article selection process

As eligibility criteria (inclusion and exclusion), only Systematic Reviews^1^ (as outlined by the acronym PECOs were included):

P (Population): Children or Adolescents

E (Exposure): nutritional status (Obesity and/or overweight and/or underweight)

C (Comparison): nutritional status (Normal/Ideal)

O (Outcome): Dental caries experience

S (Study design): Systematic reviews with or without meta-analysis

Studies were excluded according to the following criteria: outside the proposed theme, other types of study design, animal or *in vitro* studies, no direct comparison between nutritional status and dental caries experiences, adult population, and dental caries resulting from weight status.

The selecting process was performed by three researchers (TOF, PAC, and LAAA). A consensus was reached after discussion on every discrepancy between the examiners. To evaluate agreement between authors, 10% of the publications were randomly selected, their classification was compared, and a Kappa statistical index was determined (0.80). Studies were independently chosen by two researchers (TOF and PAC). If this process showed any disagreement of opinions, a third reviewer (LAAA) was consulted to resolve it. When titles and abstracts were unclear, the studies were read in full and our eligibility criteria was reapplied as previously described.

### Synthesis of narrative data (Data extraction)

The relevant data was independently extracted by two independently (TOF and PAC) and detailed by year of publication. The data of the selected articles were allocated and organized as follow:

- The methodological data of the selected SR were organized in tables, including author; year; search period; total number of studies (after duplicate removal); included studies; first and last year of the studies; scientific journal of publication; objective; design; PECO; type of included studies; electronic search bases; manual search in clinical study records and gray literature; assessment of methodological quality or risk of bias in primary studies; evaluation of evidence certainty; checklist of a writing guide; evaluation of publication bias; and protocol registration.
- Data of interest were organized in tables including the age group of the sample, number of participants, dental caries index, nutrition assessment index, and relation between dental caries and nutritional status.
- Overlapping data across the included systematic reviews included the evaluation of the overlapping of a primary study across the assessed systematic reviews using Excel^®^ during data extraction. The corrected covered area (CCA) was calculated and considered in the results. For interpretation, a CCA lower than 5% was considered a slight overlap; 6-10%, moderate; 11-15%, high; above 15%, very high.^13^ The number of included systematic reviews (c), number of index publications (r), number of total primary studies — including double counts (N) — were inserted into the *CCA formula:* (N−r)/(rC−r) .^2^

### Synthesis of narrative data (Methodological quality assessment/ risk of bias)

The risk of bias of the included systematic reviews was independently evaluated by two researchers (TOF and PAC), based on the ROBIS tool, which consists of three phases: (1) Assessment of relevance, (2) concerns about the review process, and (3) Judgment of risk of bias.^14^ In the first phase, whether the review guiding question was appropriate to the objective of a study was evaluated, answered as Yes, probably yes, probably no, no, or no information. In the second phase, each criterion of eligibility, identification and selection of studies, data extraction, a critical evaluation of each study, and the findings of the synthesis were assessed. The third phase involved classifying the reviews according to the risk of bias in phase 2, in which the final classification of the risk of bias was considered [high, low, or uncertain].

The methodological quality of the included systematic reviews was critically evaluated by two independent reviewers (TOF and PAC) using the AMSTAR 2 tool, an updated and reliable tool consisting of 16 items, seven of which are considered “critical areas.” Each item was evaluated using the following “Yes”, “No,” or “Partially yes” scores to evaluate the methodological quality of the assessed systematic reviews.^3^ “Yes” indicates a positive result, “No” refers to items that we were unable to evaluate due teo lack of information, and “Partial Yes” indicates a partial adherence to the norm.

### Synthesis of quantitative data (meta-analysis and certainty of the evidence)

The data of the SR meta-analysis were synthesized. Data on the software used to estimate and create forest plots, heterogeneity, general effect test (random or fixed), p values, and confidence intervals were obtained.

Based on each evaluated outcome, certainty of the evidence, using the Grading of Recommendations Assessment, Development and Evaluation (GRADE) approach, was assessed. The presence of serious or very serious issues related to risk of bias, inconsistency, indirectness, imprecision, and publication biases were evaluated. ^4^

## Results

### Search Strategy and article selection process

We found 278 studies by searching Medline via PubMed (37), Web of Science (95), Scopus (76), Virtual Health Library (LILACS) (2), Cochrane library (4), and Embase (64) databases. After removing duplicates, we screened 119 SR. Of these, we excluded 96 as their titles and abstracts failed to meet our eligibility criteria. We evaluated the full texts of 23 SR. After full reading, we excluded five studies for the reasons in Supplemental Table 2. After searching the electronic databases, we evaluated SR registration bases such as PROSPERO and Open Science Framework, the Epistemonikos database (in which we assessed the 100 first findings), manually assessed the reference lists of the selected SR, and included one additional SR. We also searched the gray literature (the 100 first findings on Open Grey and Google scholar), finding no additional studies. Our final selection included 19 SR^8,9,10,17-32^ in our analysis (Supplemental Figure 1).

### Synthesis of narrative data (Data extraction)

#### Methodological aspects of the included SR.

Among the included studies, eight^10,17,19,21,22,24,25,9^ focused on the association between dental caries experiences and nutritional status; 10^18,8,20,26-29,30-32^, on dental caries and obesity and/or overweight; and one^23^, only on the association between underweight children and teenagers and dental caries experiences. Our assessment of information sources for all included SR showed that studies used Medline/PubMed the most. According to the Cochrane handbook for Systematic Reviews, additional searches are an extremely important to add relevant studies to SRs, including the gray literature and reference lists, for example. We found that only four SR failed to perform this search.^9,21,25,27^ We observed that four studies failed to correctly report an evaluation of methodological quality since two ignored this procedure^8,20^, one^25^ used a reporting guide to evaluate quality of evidence, and another failed to report the used tool.^30^ Most SR^18,20-26,28^ reported following the PRISMA checklist but only four^18,21,22,32^ had registered their protocol records. Moreover, only three studies^18,22,23^ reported assessing certainty of evidence by GRADE (Supplemental Table 3).

#### Main findings of interest in the selected SR

We assessed the number of included SR (c=19), index publications (r=175), total primary studies — including double counts (N=294) — by a matrix of evidence in Excel^®^. Our corrected covered area calculation considered the primary study overlap across the included SR as very high (CCA=37%). (Supplemental Figure 2)

The first included SR was published in 2006^8^ and the most recent one, in 2022.^30^ The SR we evaluated analyzed studies published from 1989 (first included study) to 2020 (last included studies). The age of the patients included in the studies ranged from 0 to 20 years, whereas the number of participants, from 3.246^23^ to 98.349.^24^ The most used dental caries index was DMFT/DMFS for permanent dentition^9,10,17,19,22,24-32^ and the dmft/dmfs for primary dentition^9,10,17,18,19,21-26,28,29,31,32^. Only one study reported using the ICDAS scoring method.^18^ All included SR reported body mass indices as the used nutritional status index. We observed the association between dental caries and obesity/overweight in seven SR^17,20-22,28,30^, one between dental caries and underweight^23^, and 11 found no association.^8-10,19,24-27,29,31,32^(Table 1).

**Table 1:** Data extraction: Characterization of the included studies

Author (Year)	Period of search	First and last evaluated year of the chosen studies	Sample age	Number of participants	Dental caries index	Nutrition assessment index	Presence of association
Aceves-Martins, et al.^32^ (2022)	1995-2021	2005-2019	0-18 years	17.312	(DMFT, (DMFS, deft, ICDAS)	BMI	Inconclusive
Silveira, et al.^31^ (2022)	Up to 20th June, 2020	2010-2019	12mo-18 years	7.542	(Dmft, DMFT, dmfs, DMFS, deft, defs)	BMI	Inconclusive
Singh, et al.^30^(2022)	1990-2020	Not reported	2-20 years	Not reported	dmft, deft	BMI	Yes
Alotaibi, et al.^20^ (2020)	2007-2020	2007-2020	mean age (8.5 years)	6.886	MD	BMI	Yes
Alshehri, et al.^21^ (2020)	Not reported No time of publication limit was placed	2014-2019	6-17 years	4.562	DMFS/dmfs, DMFT/dmft, DFT, DT	BMI	Yes
Chanchala, et al.^17^ (2020)	2009-2019	2007-2019	2.5-18 years	45.722	DFT/DMFT; dft/dmft	BMI	Yes
Manohar, et al.^18^ (2020)	Up to 28th February of 2019	2007-2016	<6 years	4.892	dmft/ dmfs, defs, dfs index, ICDAS scoring method	BMI	Yes
Singh and Purohit^23^ (2020)	Up to January 2020.	1993-2018	6 months-19 years	3.246	DMFT/dmft	BMI	Yes
Angelopoulou, et al.^22^ (2019)	Up to 2018	1982 - 2017	5 months- 6 years mean age was 4.22 years.	21.351	dmft/dmfs	BMI	Yes
Paisi, et al.^24^ (2019)	1980-2014	1998-2014	0-18 years	98.349	DMFT/dmft, DMFS/dmfs, deft/defs	BMI	No
Chen, et al.^10^ (2018)	Up to March of 2017	2007-2016	< 18 years	43.860	DMFT/deft; dmfs/defs	BMI	No
Shivakumar, et al.^19^ (2018)	2005 -2016	2007-2015	2.5-17 years	8.550	DMFT/deft index	BMI	No
Li, et al.^25^ (2015)	Up to February of 2014	1995-2014	<18 years	11.625	DMFT/dmft, DMFS/dmfs, DFT, DT, deft	BMI	No
González Martínez, et al.^27^ (2014)	Up to November of 2011	2007-2011	1-18 years	23.048	DMFT	BMI	No
González Muñoz^26^ (2013)	2007- 2011	2007-2011	0-18 years	46.838	DMFT, DMFS; dmft, dmfs.	BMI	No
Hayden, et al.^28^ (2013)	1980-2010	1998-2009	<18 years	35.251	DFT, DMFT, DMFS, DEFT, DFES	BMI	Yes
Silva, et al.^29^(2013)	2005-2012	2006-2012	6-20 years	22.641	DMFT; DMFS; dmft; dmfs	BMI	No
Hooley, et al.^9^ (2012)	2004-2011	2004-2011	0-18 years	75.140	DMFT/dmft.	BMI	No
Kantovitz, et al.^8^ (2006)	1984-2004	1989-2004	02-15 years	10.908	DFT/DFS; dft/dfs	BMI	No

Legend: MD= missing data; BMI = Body mass index; DT=(number of permanent decayed teeth); DMFS/dmfs =(decayed, missing, and filled surfaces), DMFT/dmft =(decayed, missing, and filled teeth); DFT/dft = (decayed and filled teeth), dfs = (number of decayed and filled primary teeth surfaces), deft = decayed, extracted, or filled teeth ; defs = decayed, extracted, or filled surface ; decayed =(Dd), missing =(Mm), filled= (Ff) surface (Ss) or teeth (Tt) index, ECC =Early childhood caries, ICDAS = International Caries Detection and Assessment System”; International Obesity Task Force (IOTF).

Supplemental Table 4 and Supplemental Table 5 shows additional information about period of search and details of the included studies in SRs and the main findings of the selected SR selected, respectively.

Regarding secondary findings, Alotaibi, et al.^20^(2020) showed an increase in low socioeconomic standard in the caries group when compared to caries-free group. Alshehri, et al.^21^(2020) suggest that the relation between obesity and dental caries may be due to overconsumption of processed foods. Subgroup analysis in Chen, et al.^10^ (2018) showed that overweight and obese children in high-income countries tended to have more dental caries than normal-weight children’s primary and permanent teeth (Supplemental Table 5).

#### Synthesis of narrative data (Risk of Bias and Quality assessment)

We found that 17 SR showed “high risk of bias” ^8-10,17,19,20-22,24-32^ and two, “low risk of bias”^18,23^ according to ROBIS. Most problems related to phase 2 as most concerns regarded methods to find and/or select studies (domain 2), and synthesis and findings (domain 4). Regarding domain 2, the main limitation of the SRs related to the adequacy of the terms and search strategy to obtain the largest number of studies as the evaluated reviews often showed neither their entire search strategy nor all the terms used in sufficient detail to enable replication or had either inadequate combinations of controlled terms (MeSH for Medline) and keywords in their titles and abstracts and/or inadequate filters.^10,19,17,20-22,27,30-32^ On domain 4, their main limitation related to a lack of discussion of the biases of primary studies in their syntheses as they neither evaluated risk of bias using appropriate criteria^20,26^ nor considered bias in their results/conclusions ^8,9,19,17,21,23-25,27,29,30^ (Table 2). Supplemental Table 6 shows additional information about our ROBIS assessment.

**Table 2:** Assessment of risk of bias and methodological quality

	ROBIS	AMSTAR 2
Aceves-Martins, et al.^32^(2022)	High	Critically Low
Silveira, et al.^31^(2022)	High	Critically Low
Singh, et al.^30^ (2022)	High	Critically Low
Alotaib, et al.^20^ (2020)	High	Critically Low
Alshehri, et al.^21^ (2020)	High	Critically Low
Chanchala, et al.^17^ (2020)	High	Critically Low
Manohar, et al.^18^ (2020)	Low	Low
Singh and Purohit^23^ (2020	Low	Critically Low
Angelopoulou, et al.^22^ (2019)	High	Low
Paisi, et al.^24^ (2019)	High	Critically Low
Chen, et al.^10^ (2018	High	Critically Low
Shivakumar, et al.^19^ (2018)	High	Critically Low
Li, et al.^25^ (2015)	High	Critically Low
González Martínez, et al.^27^ (2014)	High	Critically Low
Gonzalez Muñoz^26^ (2013)	High	Critically Low
Hayden, et al.^28^ (2013)	High	Critically Low
Silva, et al.^29^ (2013)	High	Critically Low
Hooley, et al.^9^ (2012)	High	Critically Low
Kantovitz, et al.^8^ (2006)	High	Critically Low

Considering quality assessment by AMSTAR-2, we classified 17 studies^8-10,19,17,20,21,23-32^ as having a critically low quality due to more than one critical failure with or without non-critical weaknesses. We rated two studies^18,22^ as “low quality” due to one critical flaw (domain 7) with or without non-critical weaknesses. The critical domain 2 (protocol registration before the review) in AMSTAR-2 was a source of problem in 13 studies^8-10,17,19,20,25-31^. Only one study showed no problems in critical domain 7 (List of excluded studies and justification for exclusion),^9^ which represents justification for excluding individual studies in an SR. The concerns of domain 13 (consideration of risk of bias when interpreting results) represented a problem in 13 studies^8-10,17,19,20,23-27,29,30^ (Table 2). Supplemental Table 7 shows additional information about our AMSTAR-2 assessment.

#### Synthesis of quantitative data (Meta-analysis and certainty of evidence)

Only eight SR^10,18,20,22,23,28,30,32^ in this overview contained meta-analyses. We gathered and interpreted their outcome data to summarize their quantitative evidence.

Concerning “normal weight vs. obesity” in children and adolescents, one meta-analysis showed this association^20^ as more obese children under six years of age had dental caries. Results showed that children with obesity had a higher dental caries experience than normal-weight children.^18^ By evaluating “underweight vs, normal weight” in children and teenagers, Singh and Purohit^23^ (2020) suggested that malnourished children were significantly associated with a higher presence of dental caries after assessing 11 studies, cohort studies, and permanent dentition. Regarding “overweight vs normal weight,” Singh and Purohit’s^24^(2020) meta-analysis showed such an association by assessing dental caries according to the dmft index. In this study, children with higher weight had greater risk of having early childhood caries (ECC). When interpreting “normal weight vs. overweight/obesity combined”, Hayden, et al.^28^ (2013) found a significant association between childhood obesity and dental caries after assessing 14 studies and using body mass indices (BMI) and the international standards for child obesity (IOF) as indices. Manohar, et al.^18^ (2020) showed that the highest BMI scores had higher dental caries experience (Table 3). Assessing the relation between DMFT and obesity/overweight showed no association between them.^32^

**Table 3:** Data extraction: Meta-analysis and evaluation of certainty of the evidence

Author (year)	Age (years)	Outcome 1	Outcome 2	Outcome 3	Results of outcome 1	Results of outcome 2	Results of outcome 3	Conclusion	Certainty of evidence
Aceves Martins, et al.^32^ (2022)	-	NW x OB	NW x OW/OB (DMFT)	-	(I^2^=78.36%, P=0.3329) (MD:0.16, 95% CI=−0.16,0.48)	(I^2^=82.21%, P=0.3649) (MD=0.12, 95% CI=−0.14, 0.39)	-	Outcome 1: No association Outcome 2: No association	⨁◯◯◯ Very Low
Alotaibi, et al.2^0^ (2020)	3-14	NW x OB	-	-	(I^2^=84%, P < 0.001) (OR=2.12; 95% CI: 1.17 to 3.87, p=0.014)	-	-	Increases obesity in the caries group	⨁◯◯◯ Very Low
Manohar, et al.^18^ (2020)	<6	NW x OW	NW x OB	NW x OB/OW	Cohort design: I^2^: 0% (PR=1.36, 95% CI: 0.97 to 1.90; p<0.05. Case-control design: (OR=1.09, 95% CI, 0.64 to 1.85, p=0.75).	Cohort design : (PR=1.45, 95% CI: 1.13 to 1.85, p=0.003). Case-control design: (I^2^=77.7%, p=0.011 OR=1.57, 95% CI, 0.60 to 4.15, p=0.361).	Cohort design: (I^2^=0%, p=0.393) PR=1.29, 95% CI: 1.03 to1.61; p=0.025). Case-control design: (I^2^=79.8%, p=0.007) (OR=1.49; 95% CI: 0.68 to 3.25; p=0.317).	Outcome 1: No association. . Outcome 2: Children with obesity have a higher dental caries experience than those with normal weight. Outcome 3: Highest BMI scores had higher dental caries experiences	⨁◯◯◯ Very Low
Singh e Purohit^23^ (2020)	6-19	NW x UW	NW x OW	NW x OW And NW x OB	All 11 studies: (I^2^=84%, p< 0.001 OR=1.96, 95% CI, 1.23 to 3.12, p< 0.001). Cohort design: (I^2^=0%, p=0.54 OR=2.69, 95% CI, 2.05 to 3.53, p<0.001). Permanent dentition: (I^2^=44%, p=0.15 OR=3.56, 95% CI, 2.21 to 5.74, p<0.001). Primary dentition: (I^2^=86%, p< 0.001 OR=1.45, 95% CI, 0.78 to 2.69, p=0.24). Early childhood caries: (I^2^=86%, p<0.001 OR=1.67, 95% CI, 0.88 to 3.17, p=0.12). Dmft index: (I^2^=32%, p=0.16 Mean difference=0.45, 95% CI, 0.21 to 0.70, p=0.0003).	Dmft index: I^2^=32%, p=0.16 Mean difference=0.45, 95% CI, 0.21 to 0.70, p=0.0003). Dmfts index: (I^2^=73%, P=(not reported Mean difference=−0.23, 95% CI, −1.15 to 0.69, p=0.62).	NW x OW: Dmfts: (I^2^=62%, p=0.0007 Mean difference=−0.39, 95% CI,−0.64 to −0.14, p=0.0002). Dmfs:(I^2^=0%, p=Not reported; Mean difference=0.14, 95% CI,−0.12 to 0.41, p=0.28). NW x OB: dmft index: (I^2^=0%, p=0.74 Mean difference=−0.07, 95% CI, −0.31 to −0.17, p=0.57). dmfts: (I^2^=0%, P=not reported Mean difference=0.35, 95% CI, −0.09 to 0.79, p=0.12)	Outcome 1: All 11 studies: Malnourished children were significantly associated with a higher experience of caries. In longitudinal studies, malnourished children were significantly associated with a higher experience of caries. Permanent dentition: Malnourishment in children was associated with a higher experience of caries. Outcome 2: dmft: Children with greater weight have a greater risk of having ECC when assessed by the dmft index. Outcome 3: No association.	⨁◯◯◯ Very Low
Agelopoulou, et al.^22^ (2019)	5m-6	NW x UW	NW x OW	NW x OB	Primary teeth: Dmft index: (I^2^=76%, p=0.0001. Mean difference=0.21, 95% CI, −0.01 to 0.43, p>0.05). Dmfts index: (I^2^=73.3%, p=0.023. Mean difference=0.23, 95% CI, −1.15 to 0.69, p>0.05). Permanent teeth: DMFT index: (I^2^=20.2%, P=0.263 Mean difference=−0.07, 95% CI,−0.15 to 0.01, p>0.05).	Primary teeth: Dmft index: (I^2^=81.3%, P>0.0001. Mean difference=0.07, 95% CI, −0.19 to 0.34, P >.05). dmfs index: (I^2^=0%, P=0.488 Mean difference=0.14, 95% CI, −0.12 to 0.41, p>0.05). Permanent teeth: DMFT index: (I^2^=88.4%, p>0.0001. Mean difference=−0.11, 95% CI, −0.46 to 0.25, p>0.05	Primary teeth: dmft index: (I^2^=95.5%, P<0.0001 Mean difference=0.34, 95% CI, −0.26,0.94, p>0.05). dmfts index: I^2^=0%, P=0.642 Mean difference=0.35, 95% CI, −0.09,0.79, p>0.05). Permanent teeth: DMFT index: (I^2^=87.9%, P<0.0001 Mean difference=−0.14, 95% CI, −0.64 to 0.36, p>0.05)	No association between outcomes.	⨁◯◯◯ Very Low
Chen, et al.^10^ (2018)	<18	NW x UW	NW x OW	NW x OB	Primary teeth dmft index: (I^2^=76%, P=0.0001Mean difference=0.21, 95% CI, −0.01 to 0.43, p>0.05). dmfs index: I^2^=73.3%, p=0.023 Mean difference=0.23, 95% CI, −1.15 to 0.69, p>0.05). Permanent teeth DMFT index: (I^2^=20.2%, p=0.263 Mean difference=−0.07, 95% CI, −0.15 to 0.01, p>0.05).	Primary teeth dmft index: (I^2^=81.3%, p<0.0001 Mean difference=0.07, 95% CI, −0.19 to 0.34, P >.05) dmfs index: (I^2^=0%, p=0.488 Mean difference=0.14, 95% CI, −0.12 to 0.41, p>0.05). Permanent teeth (I^2^= 88.4%, P<0.0001 Mean difference=−0.11, 95% CI, −0.46 to 0.25, p>0.05).	Primary teeth dmft index: (I^2^=95.5%, p<0.0001. Mean difference=0.34, 95% CI, −0.26 to 0.94, p>0.05). dmfs index: (I^2^=0%, P=0.642 Mean difference=0.35, 95% CI, −0.09 to 0.79, p>0.05). Permanent teeth DMFT index: (I^2^=87.9%, p<0.0001 Mean difference=−0.14, 95% CI, −0.64 to 0.36, p>0.05)	No association between outcomes.	⨁◯◯◯ Very Low
Hayden, et al.^28^ (2013)	<18	NW x OB/OW	NW x OB/OW (Not considering age)	-	All 14 studies: (I^2^: Not reported, SDM = 0.104, 95% CI, − 0.001 to 0.206, p=0.049). Permanent teeth: (I^2^: Not reported) SDM = 0.124, 95% CI, −0.053, 0.301, p=0.170). Primary teeth: (I:Not reported) SDM=0.093, 95% CI, − .03 to 0.22, p=0.149)	BMI: I^2^(Not reported) SDM=0.189, 95% CI, 0.060 to 0.318, p=0.004 IOFT cut offs: (I^2^: Not reported) SDM = 0.104, 95% CI, 0.028 to 0.180, p=0.008).	-	Outcome 1: A significant association between obesity and caries. Outcome 2: A significant association between caries and obesity was found in the studies using standardized measures to assess child obesity such as BMI-for-age centiles. A significant association between caries and obesity was observed in the studies using standardized measures to assess child obesity such as IOFT cut off for-age centiles.	⨁◯◯◯ Very Low

Footonotes: OB- obesity, OW- overweight, UW- underweight, NW- Normal weight; LBMI - Lower BMI; IOFT -International standards for child obesity; BMI - Body mass index; ECC- Early childhood caries; SDM- Standard difference means, DMFT- decayed, missing, and filled teeth (permanent), dmft- decayed, missing, and filled teeth (primary), dmfs-decayed, missing, and filled surfaces.

## Certainty of the evidence

Our GRADE assessment showed a very low certainty of evidence for the included meta-analyses (Table 3).^10,18,20,22,28,32^Supplemental Table 8 shows additional information on our GRADE assessment.

## Discussion

Overviews are increasingly important forms of evidence synthesis as they summarize research from results of multiple SR.^12^ This overview evaluated the quality of the chosen SRs by AMSTAR-2 and their risk of bias by the ROBIS 2 checklist (including their accordance with PRIOR).^12^ Our method shows the quality of their results and conclusions. Our narrative results suggest that children and adolescents’ nutritional status differences fail to influence dental caries experiences. Of the 19 included SR, 11 suggested no association between these variables.^8-10,19,25-28,30,32,31^

Chen, et al.^5^ (2018) investigated the association between permanent and primary dentition caries and BMI in children, showing no significant associations. However, Singh and Purohit^6^ (2020) found that underweight children were associated with a higher prevalence of dental caries. In addition to this assessment of children aged under six years, Manohar, et al.^18^ (2020) indicated that children with higher BMI scores (overweight/obesity) had more dental caries experiences, results that resembled those for undernutrition in children of the same age.^23^ The results of some SR in children and teenagers aged from 0 to 20 years suggested an association between high BMI (overweight and obesity) and dental caries.^17,20-22,28,30^ Alshehri, et al.^21^ (2020) included only studies performed in Kingdom of Saudi Arabia populations and strengthened the need for public health initiatives. Chanchala, et al.^17^(2020) found a slight majority of studies suggesting an association between high BMI and mixed dentition caries but their discussion explains that universally combining the results of their primary universally would provide inconsistent results. Thus, they deemed further research necessary to reduce limitations and offer conclusions about BMI and dental caries. Several SR highlighted dietary habits, including high ingestion of complex carbohydrates, as their main hypothesis for the association between dental caries and higher BMI.^8,9,17,25-27,32^ They also discussed parents’ literacy, socioeconomic status, and local conditions.^17,26,27,32^Regarding lower BMI scores, hypotheses related to lower knowledge about general and oral hygiene due to socioeconomic inequalities as poverty also enhances the risk of malnutrition, increasing healthcare costs.^23^

The assessed SRs should have offered an interesting discussion on the indices their primary studies used to access dental caries experience. Some recognize the detection of initial caries and others underestimate caries scores by only quantifying them (rather than offering complete information on caries development). Attempts to establish a standard in dental caries assessment are very important^18^ but discrepant diagnoses in research remain a source of limitations. Their great heterogeneity, absence of suitable sample sizes, and a lack of unification of dental caries indices may interfere with the assessment of primary studies, resulting in results without no associations or inconclusive ones and a high probability of unsupported conclusions due to their methods. We may the same about nutritional status.

Our results suggest a careful analysis concerning three important methodological evaluations in the included SR: 1- Risk of bias; 2- quality assessment; and 3- certainty of evidence. The risk of bias assessment two reviewers independently performed on ROBIS classified most included SR as having a “high risk of bias” stemming from systematic weaknesses, design limitations, or review management or analysis altering results. This can negatively affect the relevance of the evidence. Concerning quality assessment, AMSTAR 2 was an appropriate choice for this overview due as it provides a wide assessment of the quality and flaws that may have occurred during an SR. We classified two studies^18,22^ as having a “low quality” due to a critical flaw (domain 7) with or without non-critical weaknesses but these were the minority in the overview since the remaining 17 studies ^8-10,17,19,20,12,23-32^ showed “critically low quality” due to more than one critical failure with or without non-critical weaknesses. We must consider this fact during the interpretation of the results in the selected SR.

Considering SR outcomes, we found their certainty of evidence to be very low, in which “inconsistency” caused problems in all evaluated meta-analyses whether due to their great heterogeneity, absence of interpretation data, confidence intervals with minimal or no overlap, or the association of great heterogeneity and confidences intervals with minimal or no overlap. Inconsistency greatly reduces confidence in effect estimates. We strongly suspect of publication bias in four outcome evaluations,^10,24^ which introduces bias due to negative results since peer reviewers are more likely to judge approvingly if results are positive.^11^

A limitation of this overview reefers to its incorporation of low and critically low methodological quality systematic reviews, i.e., SRs with great risk of publication bias. One of the main limitations of the included studies was their lack of a fully displayed search strategy and/or no detailed description of all the used terms to enable replication. Although lack of budget and time can be a problem in some SR, this type of study depends on reproducibility. Thus, the mentioned flaws critically contribute to the reproducibility crisis in science^33,34^. Errors can also occur in the search strategies. As they are sometimes conducted in suboptimal ideal standards, this information disappears when strategies are only partially described.^11^ The absence of reports and discussions on the biases of primary studies also offers a limitation in most SR since they either fail to evaluate risk of bias by appropriate criteria or ignore it in their results/discussion. Those limitations can also introduce publication bias into an SR as the presence of bias in primary studies could possibly influence the decision of the scientific journals to publish an SR since positive results are more likely to be published than negative results. Protocol registration prior to performance is an essential and practically mandatory step for SRs. Some of the included SR shows this flaw, causing a lack of transparency in the applied method, a reduction of replication possibility, and a possible and direct implementation of selection bias.

This overview attempted to control methodological bias in its elaboration. A protocol in PROSPERO database was made and registered prior to the beginning of this overview, including appropriate eligibility criteria for our guiding question and enough details to enable their comprehension. We used a peer-reviewed search strategy before searching the databases, clearly describing our only restriction to our search based on study design (systematic reviews), which was necessary due to the nature of this research. Moreover, this overview comprised an appropriate range of databases, additional methods, and gray literature following the Cochrane handbook^1^. We have made efforts to offer our full search strategy with all terms and associations we used to search the chosen databases and enable replication. We applied no restrictions on date, language, or publication format to our search strategy to achieve the largest number of eligible studies in the literature. During title and abstract screening and full-text studies assessment for inclusion, we tried to minimize errors that might have stemmed from two independent reviewers screening, assessing full texts, and including SRs. We made the same efforts during our data collection and risk of bias and methodological quality assessment. We collected sufficient information on study characteristics, gathered results, and showing them in this overview to enable the appropriate analysis of heterogeneity, synthesis, and result applicability. Our narrative synthesis respected the nature and similarities of the included SR. During result and conclusion interpretation, we assessed bias and methodological quality in the chosen, addressing and incorporating them into our judgment.

The general society benefits from the information of this overview as it may better guide public health strategies since nutrition and oral health are major sources of public investment, configuring an extensive variety of studies published by the scientific community. Therefore, our results can help to guide such investment. Moreover, the knowledge regarding the lack of association between those variables will enable clinicians to prepare personalized treatment plans based on solid scientific literature, contributing to a better practice.

We believe in a strong demand for future research on this topic to improve the methodological quality of studies since we currently have several tools that could be used to increase the level of evidence of SRs and thus minimize potential risks of bias, providing greater security and assertiveness for clinicians’ decision-making. More studies being conducted with greater methodological rigor and a variety of measurement indices other than BMI will enable the elaboration of a new overview, providing room for authors to address broader guiding questions than those evaluated in individual SRs, synthesizing the best evidence, generating greater certainty for clinicians, and improving public health policies. Our results and discussion directly affect the future of research on the topic since our findings enabled good suggestions, explain the decision-making process of methodological aspects, and influence the assessment of risk factors of future research.

## Conclusion

As the included SR had high risk of bias, low methodological quality, and very low certainty of evidence, most found no association between nutritional status and dental caries experiences in children and teenagers. Regardless of the quantitative results for the specific outcomes we summarized in this overview, interpreting their conclusions requires careful analysis to guide public policies and clinicians’ judgment.
